# Protamine protects against vancomycin-induced kidney injury

**DOI:** 10.1128/aac.01236-24

**Published:** 2025-01-17

**Authors:** Justin Shiau, Patti Engel, Mark Olsen, Gwendolyn Pais, Jack Chang, Marc H. Scheetz

**Affiliations:** 1 Department of Pharmacy Practice, College of Pharmacy, Midwestern University 69281 , Downers Grove, Illinois, USA; 2 Department of Pharmacy, Northwestern Memorial Hospital 24560 , Chicago, Illinois, USA; 3 College of Pharmacy, Pharmacometrics Center of Excellence, Midwestern University 24560 , Downers Grove, Illinois, USA; 4 Department of Pharmaceutical Sciences, College of Pharmacy, Midwestern University 15474 , Glendale, Arizona, USA; 5 Department of Pharmacology, College of Graduate Studies, Midwestern University 69281 , Downers Grove, Illinois, USA; Columbia University Irving Medical Center, New York, New York, USA

**Keywords:** kidney injury, megalin, nephroprotection

## Abstract

Vancomycin causes kidney injury by accumulating in the proximal tubule, likely mediated by megalin uptake. Protamine is a putative megalin inhibitor that shares binding sites with heparin and is approved for the treatment of heparin overdose. We employed a well-characterized Sprague-Dawley rat model to assess kidney injury and function in animals that received vancomycin, protamine alone, or vancomycin plus protamine over 5 days. Urinary KIM-1 was used as the primary measure for kidney injury, while plasma iohexol clearance was calculated to assess kidney function. Animals had samples drawn pre-treatment in order to serve as their own controls. Additionally, since protamine is not a known nephrotoxin, the protamine group also served as a control. Cellular inhibition studies were performed to assess the ability of protamine to inhibit organic anion transporter (OAT1 and OAT3) and organic cation transporter-2 (OCT2). Rats that received vancomycin alone had significantly increased urinary KIM-1 on day 2 (24.9 ng/24 h, 95% CI 1.87–48.0) compared to the protamine alone group. By day 4, animals that received protamine with their vancomycin had KIM-1 amounts that were elevated compared to protamine alone as a base comparison (KIM-1 29.0 ng/24 h, 95% CI 5.0–53.0). No statistically observed differences were identified for iohexol clearance changes between drug groups or when comparing clearance change from baseline (*P* > 0.05). No substantial inhibition of OAT1, OAT3, or OCT2 was observed with protamine. IC_50_ values for protamine were 0.1 mM for OAT1 and OAT3 and 0.043 mM for OCT2. Protamine, when added to vancomycin therapy, delays vancomycin-induced kidney injury as defined by urinary KIM-1 in the rat model by 1–3 days. Protamine putatively acts through the blockade of megalin and does not appear to have significant inhibition on OAT1, OAT3, or OCT2. Since protamine is an approved FDA medication, it has clinical potential as a therapeutic to reduce vancomycin-related kidney injury; however, greater utility may be found by pursuing compounds with fewer adverse event liabilities.

## INTRODUCTION

Vancomycin is one of the most widely used antibiotics within United States in-patient healthcare systems, accounting for 11.9% of all antibiotic use for inpatient antimicrobials (1,258 out of 10,612) in a 2015 hospital survey (1). As a tricyclic glycopeptide, it is primarily indicated for severe Gram-positive bacterial infections, including methicillin-resistant *Staphylococcus aureus* (MRSA) (2). For about 70 years now, vancomycin has remained a first-line antibiotic widely used and studied across people of all ages. Despite its efficacy in the treatment of MRSA infections, one common limitation of its use is its association with acute kidney injury (AKI) (3), otherwise known as vancomycin-associated nephrotoxicity (VAN). A systematic review and meta-analysis found that vancomycin is associated with up to 2.5 times greater risk of AKI compared to other antibiotics (4).

**Fig 1:**
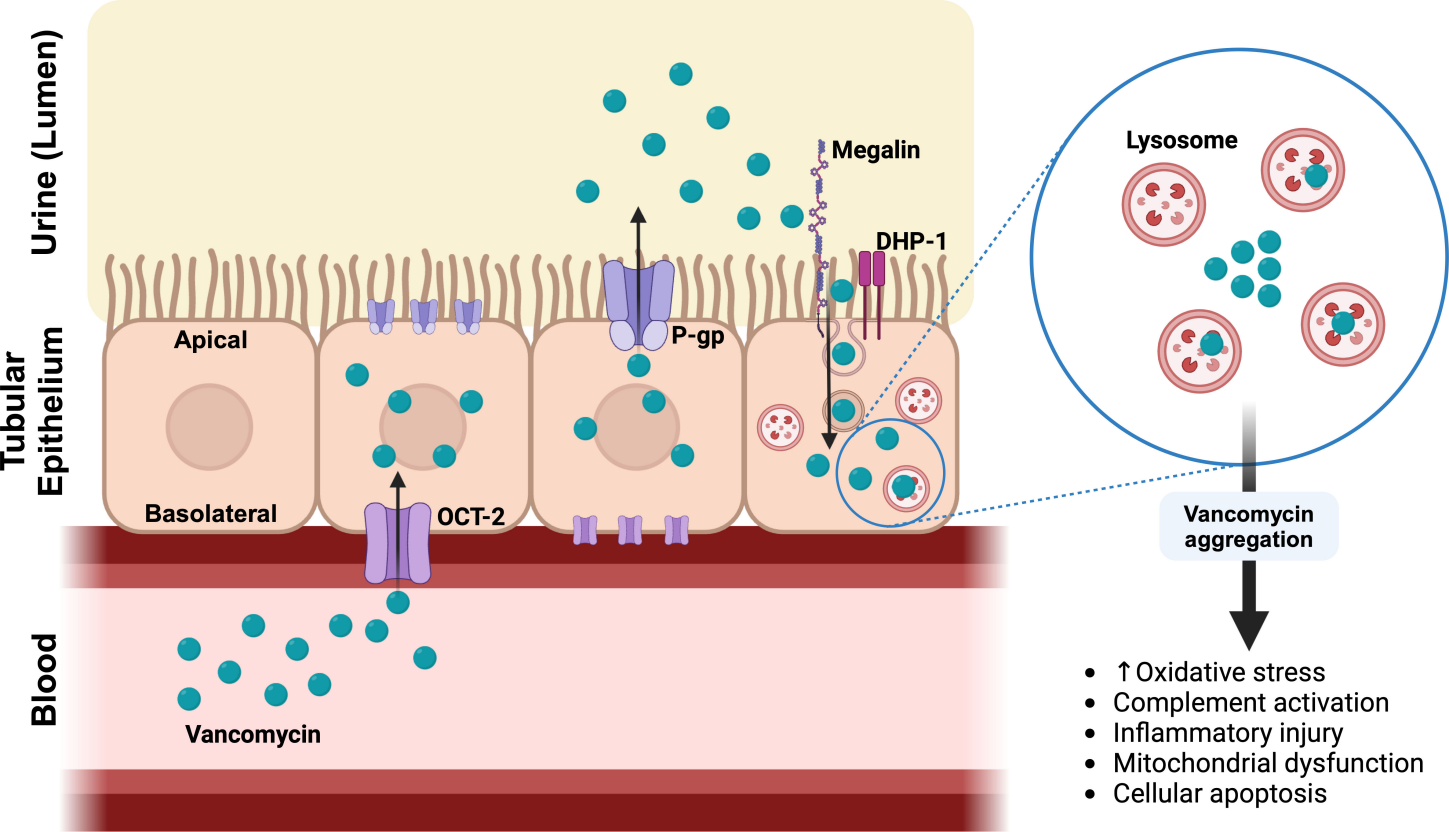
Mechanism of action of vancomycin nephrotoxicity in the renal proximal tubule cells. Created in BioRender.

VAN is attributed to two primary mechanisms, intracellular accumulation and complex formation with uromodulin and blockade of the proximal tubule (5, 6). While the individual contributions of each mechanism to the extent of kidney injury are not yet clear, intracellular accumulation is a hallmark (3, 6–8). Cellular accumulation occurs through multiple pathways both basolaterally and apically (Fig. 1) (6). Basolaterally, vancomycin is actively transported from blood circulation into kidney proximal tubule cells through the organic cation transporter (OCT)-2 located on the basolateral membrane of the tubular cell. Next, it is secreted into the tubular lumen from the apical membrane of the proximal tubule by an efflux transporter, P-glycoprotein (9, 10). Vancomycin that is in the tubular lumen (either via secretion or free filtration) is then transported across the apical membrane by apical endocytosis through dehydropeptidase-1 (DHP-1) and megalin. Intracellular vancomycin is aggregated by lysosomes leading to a cascade of oxidative stress, complement activation, inflammatory injury, mitochondrial dysfunction, and cellular apoptosis (2).

**Fig 2:**
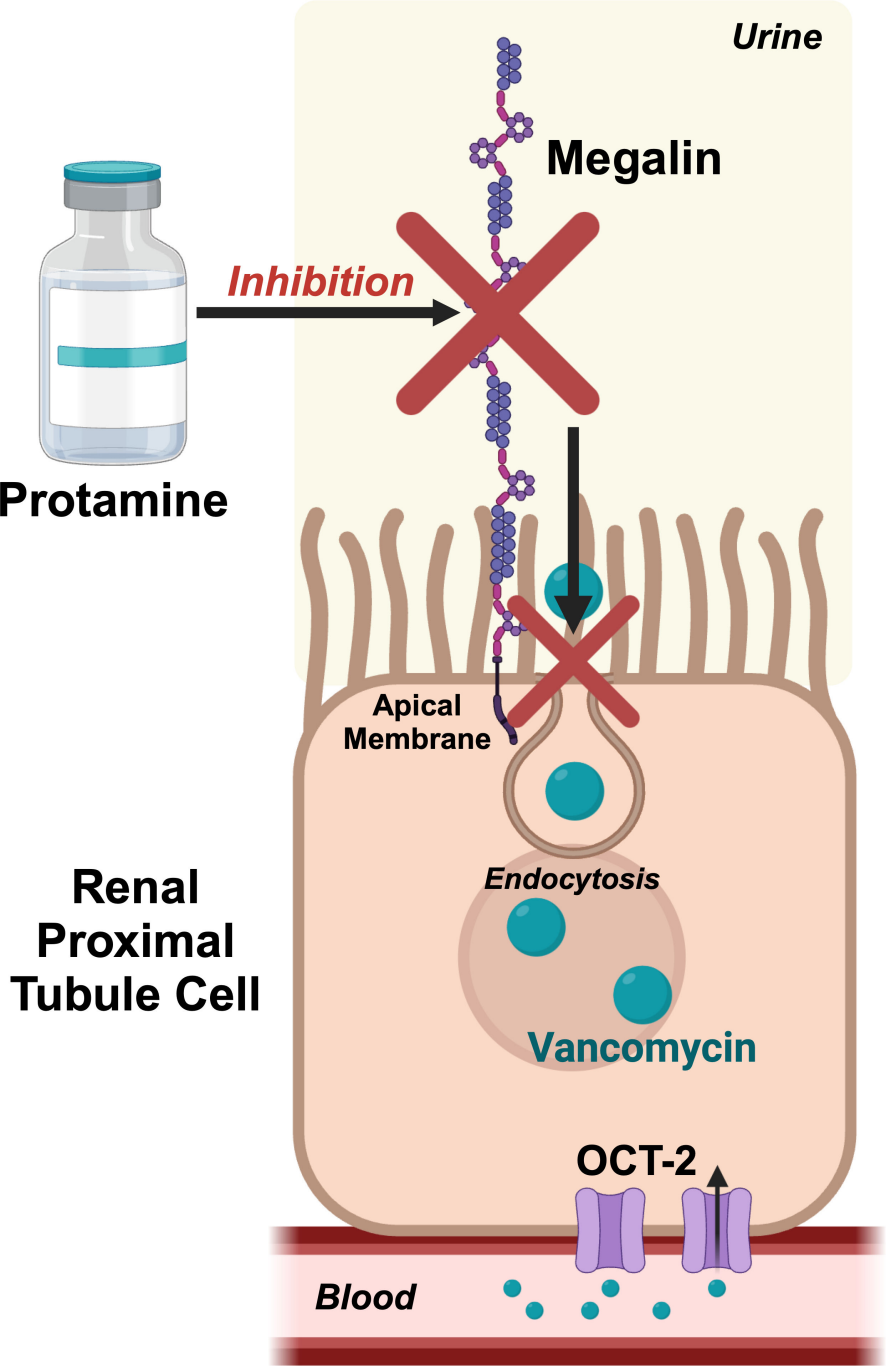
Putative mechanism of megalin inhibition by protamine and the protective effects from vancomycin nephrotoxicity. Created in BioRender.

Preventing VAN is possible via multiple pathways. First, a very clear exposure–response relationship exists (11–16). Lower area under the curve exposures result in less toxicity. Second, it is theoretically possible to prevent vancomycin from accumulating intracellularly. We (17) and others (18, 19) have previously demonstrated that administering cilastatin, a putative megalin inhibitor, reduces kidney injury (6, 18). However, the effect of cilastatin is modest, and multiple mechanisms of action, such as organic anion transporter inhibitions (20, 21), complicate mechanistic translation. While protective agents are not yet part of the therapeutic plan for agents that are toxic to the kidney, it is notable that this concept is well applied in cancer [e.g., sodium 2-mercaptoethane sulfonate (MESNA) for cyclophosphamide (22)] or N-acetylcysteine (NAC) for liver injury prevention in acetaminophen overdose (23). Megalin is a large glycoprotein expressed on the apical surface of the proximal tubule that mediates intracellular signal transduction and plays an important role in the reabsorption of glomerular-filtered substances (18, 24). Demonstrating the premise, megalin knockout mouse models have shown strong efficacy in suppressing drug-induced kidney injury (25, 26). Vancomycin binds to megalin receptors, which causes reuptake into these proximal tubule epithelial cells. Overall, transporter inhibition during active tubular secretion can have varying effects on drug concentrations (27–29). Inhibiting transporters remains an area of study that has the potential to improve outcomes for neccessary drugs that are nephrotoxic (Fig. 2).

Based on chemical structure, we hypothesized that protamine might block megalin and decrease VAN. While relatively little information is available on the megalin-binding capacity of protamine, protamine shares binding sites with heparin (which is the current target for FDA-approved use) and megalin (30, 31). Supporting this possibility of megalin blockade for the purpose of decreasing cellular accumulation and toxicity, Nagai and colleagues (32) found that protamine inhibited the uptake of gentamicin in cultured opossum kidney cells that expressed megalin and cubilin. Megalin antagonists that block drug reabsorption would be a significant advance as no drugs are approved for this indication.

## MATERIALS AND METHODS

### Experimental design and animals

**Fig 3:**
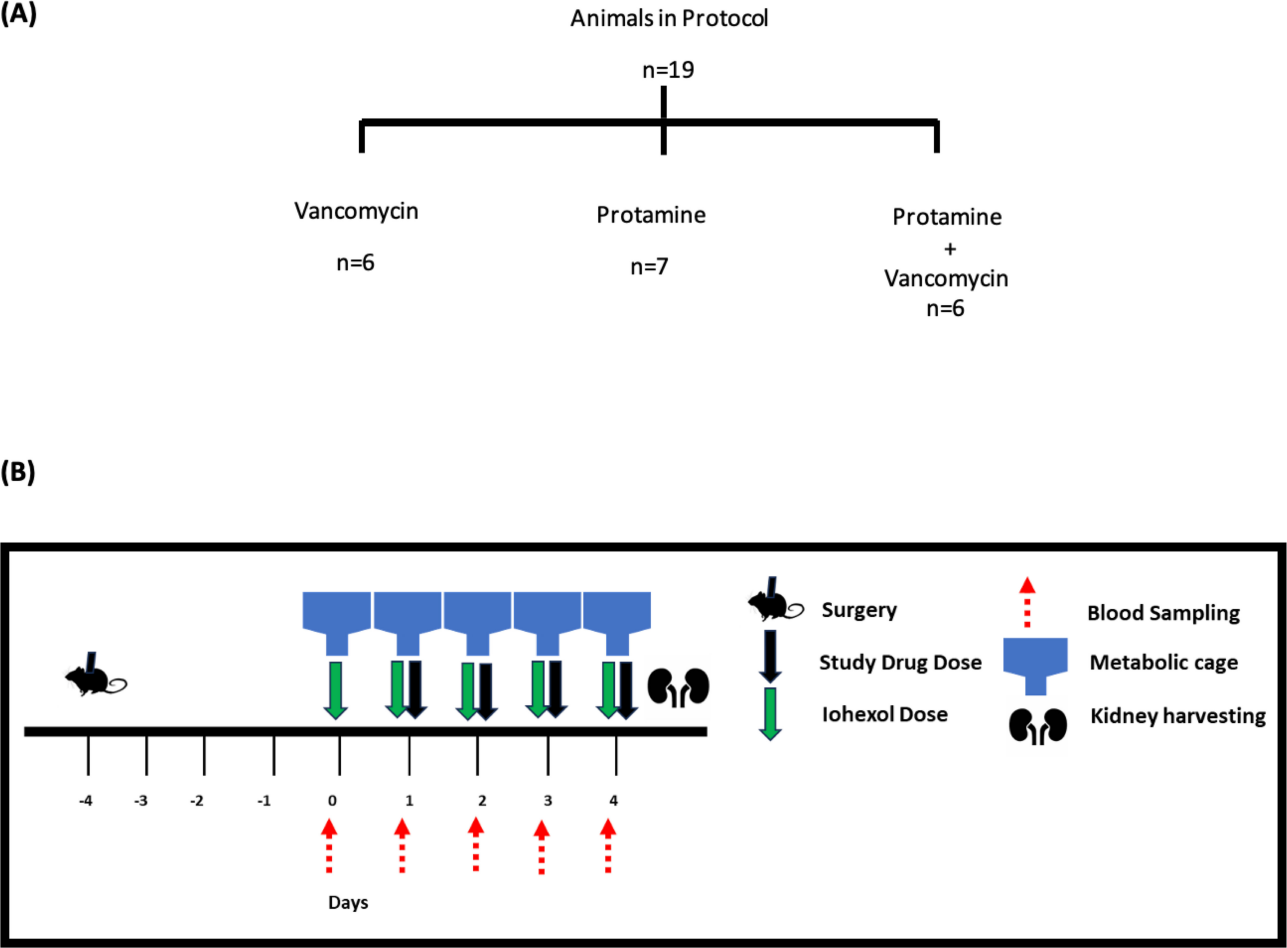
Experimental design. (**A**) Animals divided into each protocol. (**B**) Scheme indicating time flow between surgery and when animals are placed into metabolic cages for dosing (PRT and VAN), blood and urine sampling, and kidney harvest.

Male Sprague-Dawley rats (*n* = 19; age 8–10 weeks; 268–304 g; Envigo, Indianapolis, IN, USA) underwent double jugular catheterization (33) (Fig. 3A). Surgery was performed under ketamine (100 mg/kg of body weight) and xyalzine (10 mg/kg of body weight) anesthesia with buprenorphine (0.02 mg/kg of body weight) given post-operatively for pain management. Animals were allowed to recover 4 days before the administration of any drug. Animals were housed in metabolic cages starting 1 day prior to day 0 with access to food and water *ad libitum* for the duration of the study (Fig. 3B).

Animals were divided into three experimental treatment protocols. Using a dedicated jugular vein for dosing, the animals received clinical grade intravenous protamine and/or vancomycin in doses allometrically scaled to humans according to the following protocols: (i) protamine sulfate (1.5 mg/kg of body weight) and vancomycin (150 mg/kg of body weight) (*n* = 6); (ii) protamine only (1.5 mg/kg of body weight) with saline sham (sham for volume of vancomycin) (*n* = 7); or (iii) vancomycin only (150 mg/kg of body weight) with saline sham (sham for volume of protamine) (*n* = 6) (34–36). Saline sham was given to match the volumes of protamine and vancomycin doses to maintain euvolemia. On day 0, all animals received only iohexol (51.8 mg/day) intravenously to establish baseline GFR measurements. Drugs were administered as described above by treatment protocols to all groups on days 1–4. Infusion times for each of the drugs include (i) protamine sulfate, 5 minutes; (ii) vancomycin and iohexol, 2 minutes; and (iii) saline sham, 5 or 2 minutes depending on what the sham was replacing.

### Blood, urine, and kidney sampling

Blood samples were withdrawn from a designated jugular catheter. Samples were drawn at time 0 (prior to dosing) and at 30 and 60 minutes after iohexol dosing for protamine + sham and vancomycin + sham groups. Sampling time points for protamine + vancomycin group were 0, 45, and 90 minutes to account for infusion times of both drugs and iohexol in six animals. All blood samples (0.2 mL, maximum three samples/animal) were taken daily with an equivalent volume of normal saline (NS) injected to maintain euvolemia. Blood samples (with EDTA added) were centrifuged at 16,000 × *g* for 10 minutes, and the resulting plasma was aliquoted (120 µL) and stored at −80°C for batch analysis later.

Urine samples were collected daily, and volumes were measured starting from day 0 to day 4. Samples were centrifuged at 500 *g* for 10 minutes, and the resulting supernatant was aliquoted (1.5 mL) and stored at −80°C for renal biomarker analysis later.

Animals were euthanized (ketamine/xylazine) on day 4 after dosing/sampling for that day, with terminal blood and urine collected. Kidneys were harvested and rinsed in cold NS; the right kidney was flash frozen in liquid nitrogen and stored at −80°C. The left kidney was rinsed in NS and then stored in 10% buffered formalin phosphate and stored at room temperature.

### Chemicals and reagents

The agents used for treatments were clinical grade Protamine Sulfate Injection (Fresenius Kabi, Lake Zurich, IL, USA); clinical grade Vancomycin HCl (Hospira, Lake Forest, IL, USA); Omnipaque (iohexol) injection (GE Healthcare, Marlborough, MA, USA); and Normal Saline (Veterinary 0.9% sodium chloride injection USP; Abbott Laboratories, North Chicago, IL, USA). For liquid chromatography–mass spectroscopy (LC–MS) analysis, LC–MS-grade acetonitrile, methanol, and formic acid were used (VWR, Radnor, PA, USA). Analytical-grade iohexol (Sigma-Aldrich, St. Louis, MO, USA) and isotope iohexol-d_5_ (Cayman Chemicals, Ann Arbor, MI, USA) were also used. For the preparation of calibrators for standard curves, frozen aliquots of Na_2_EDTA-treated (anticoagulant), non-medicated, non-immunized plasma pooled from male Sprague-Dawley rats (BioIVT, Hicksville, NY, USA) were used. For cellular experiments, chemical-grade protamine sulfate USP (Product Number P3369, Sigma, St. Louis, MO, USA) was used.

### Clearance as calculated by iohexol

In order to describe plasma iohexol clearance, a physiological PK model was created with nonlinear mixed effects in Monolix (version 2023R1; Antony, France: Lixoft SAS, 2023). A two-compartment model was used for the samples, with fixed estimates for peripheral compartment volume (V2) and intercompartmental transfer (Q) being derived from the base two-compartment model fitting. Random effects were estimated for clearance (Cl) and central volume (V1). The treatment group was evaluated as a modifier of clearance. To capture daily clearance changes, each experimental day (Monday through Friday) was considered as a separate occasion with time-varying clearance. Clearance was calculated as an occasion every 24 h to capture the dynamic changes in renal function.

### Urinary biomarkers

Urinary samples were analyzed for KIM-1 using the Milliplex MAP Rat Kidney Toxicity Magnetic Bead Panel 1 (EMD Millipore Corporation, Charles, MO, USA) according to the manufacturer’s directions and as previously described (34, 36, 37). Briefly, neat urine aliquots were mixed with monoclonal antibody-coated magnetic microbeads (Bead Panel 1) and run on a 96-well plate, which also included a standard curve. Biomarker data were acquired, and concentrations were analyzed using Belysa Immunoassay Curve-Fitting Software V1 (MilliporeSigma, Darmstadt, Germany). The amount of urinary KIM-1 excreted over the 24-h collection period was obtained by multiplying the urinary concentration by 24-h urinary volume.

### Preparation of calibration curves in rat plasma

Stock solutions of iohexol and iohexol-d_5_ were prepared using purified water at concentrations of 1 mg/mL and 100 µg/mL, respectively. Calibrators for a standard curve were generated by diluting iohexol stock solution with water to produce concentrations ranging between 1 and 100 µg/mL.

For each standard curve calibrator, 36 µL of blank rat plasma was mixed with an iohexol dilution (4 µL each). Iohexol-d_5_ (4 µL) was then added to each at a final concentration of 10 µg/mL as the internal standard. Each standard curve calibrator was then mixed with 456 µL of 0.1% formic acid in methanol, vortexed, and centrifuged at 16,000 × *g* for 10 minutes. From the resulting supernatant, 100 µL was transferred to LC–MS vials and analyzed.

### Sample preparation

Plasma samples obtained at 30, 45, and 60 minutes were diluted 1:10 with blank rat plasma with 4 µL of iohexol-d_5_added to each. Iohexol-d_5_ was also added to the remaining rat plasma samples (40 µL, undiluted). All samples were then mixed with 0.1% formic acid in methanol, vortexed, and centrifuged at 16,000 × *g* for 10 minutes, with the resulting supernatant transferred to LC–MS vials for analysis.

### LC–MS methods

An Agilent 1260 Infinity II series liquid chromatography system was paired with an Ultivo triple quadrupole mass spectrometer (Agilent Technologies, Santa Clara, CA, USA) and used to analyze plasma samples. A Kinetex Polar C18 analytical column was used (2.6 µm, 50 × 2.1 mm, part number PRD-634714; Phenomenex), and column temperature was maintained at 25°C. Mobile phases were 0.1% formic acid in water (mobile phase A) and acetonitrile (mobile phase B). An isocratic method was used to separate analytes with solvent ratios of 94% mobile phase A–6% mobile phase B (0–0.50 minutes), 5% mobile phase A–95% mobile phase B (0.51–2.0 minutes), and 94% mobile phase A–6% mobile phase B (2.01–3.5 minutes) at a mobile phase flow rate of 0.8 mL/minute. Multiple reaction monitoring was used for analyte detection monitoring transitions of *m/z* 821.9–803.9 for iohexol and *m/z* 826.9–808.7 for iohexol-d_5_.

### Cell studies

Organic anion transporter (OAT1 and OAT3) and OCT2 inhibition studies with protamine sulfate were performed by Eurofins Scientific (St. Charles, MO, USA) as previously described (38, 39). Protamine sulfate was dissolved in DMSO to 1.0E-02 M and tested concurrently with *p*-aminohippurate 10 µM (substrate) for OAT1, ranitidine 10 µM (substrate) for OAT3, and metformin 100 µM (substrate) for OCT2. A pre-incubation time of 30 minutes was used, followed by 20 minutes of incubation with the substrate. CHOK1 cells were used in OAT1 and 3 experiments, and HEK cells were used in OCT2 experiments. The percentage of inhibition was calculated vs the percentage of control. The IC_50_ value (concentration causing a half-maximal inhibition of the control value) was determined by nonlinear regression analysis of the concentration-response curve from a Hill equation. Experiments were compared to referent IC_50_ results on file for OAT1 and 3 using probenecid and for OCT2 using verapamil as exemplar inhibitors.

### Statistics

Urinary KIM-1 concentrations and plasma iohexol clearances over time were compared with a repeated measures mixed model, fit via restricted maximum likelihood to have results comparable to the ANOVA results. An omnibus test, i.e., joint tests (multi-degree of freedom) of the interaction and main effects, which is similar to the *F* test in ANOVA, was used initially (STATA 17.0 BE, StataCorp LLC, College Station, TX, USA). Only models with significant interaction at *P* < 0.05 were selected for *post hoc* analysis (i.e., identification of the difference between treatment groups and days). Margins were calculated for a full factorial of the variables, i.e., main effects for each variable and interactions. Referent groups were pre-treatment baseline values and protamine alone as a treatment, except where noted. Secondarily, to remain agnostic to the outcome variable relationship over time (and assess treatment groups over time), locally weighted scatterplot smoothing (LOWESS) trendlines with 95% confidence intervals were generated. LOWESS graphs were produced in R version 4.2.2 using ggplot2. No data were excluded as outliers. All tests were two-tailed; a *P* < 0.05 was required for statistical significance. Graphs for cellular studies were generated using GraphPad version 9.3.1 and were fit with a log(inhibitor) vs normalized response function after log transformation.

## RESULTS

### KIM-1

**Fig 4:**
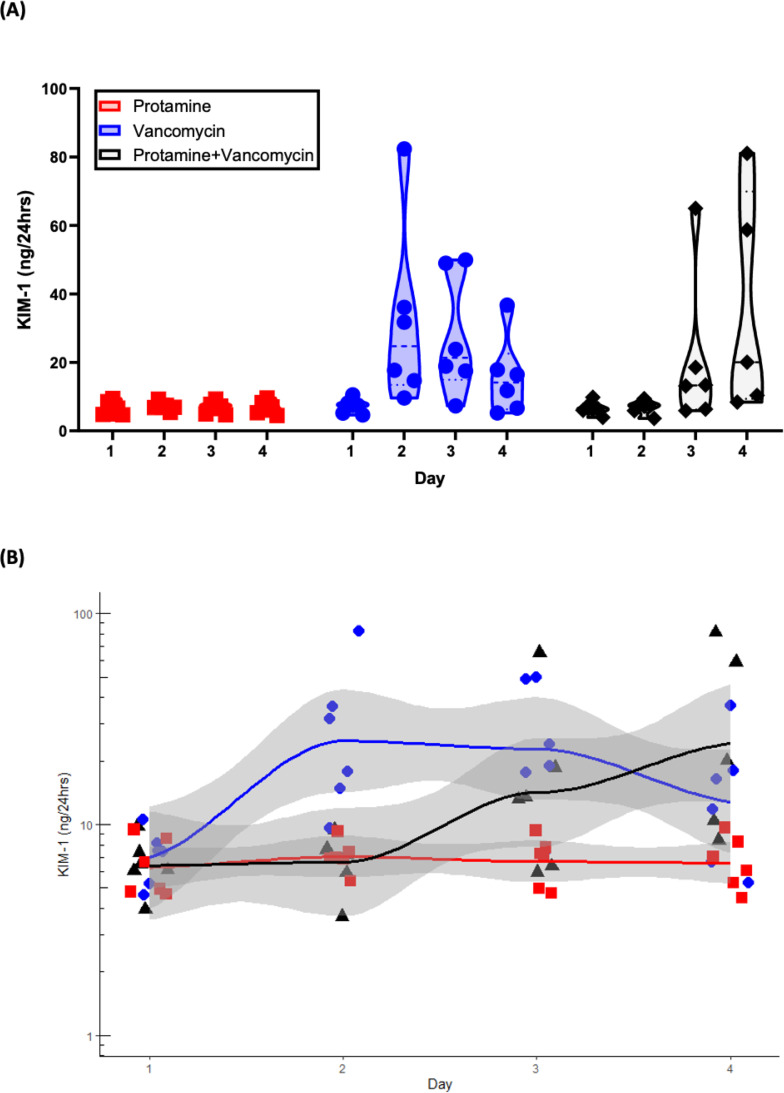
Urinary KIM-1 (ng/24 h) for each study group over study days, shown as a violin plot (**A**) and individual values with 95% CI (**B**).

The omnibus test was significant for drug group, day, and the interaction of drug and day (*P* < 0.05). Further evaluation of individual drug groups by day (with Bonferroni corrections) revealed that on day 1, prior to any drug being received, all groups had similar KIM-1 (Table 1, Fig. 4A and B). On day 2 (i.e., 24 h after drug administration), animals that received vancomycin alone had significantly increased KIM-1 (24.9 ng/24 h, 95% CI 1.87–48.0) compared to the protamine alone group as the base. Animals that received protamine did not experience this increase on day 2. On day 3, animals that received protamine had a measured KIM-1 that was numerically similar to animals that received vancomycin alone. By day 4, animals that received protamine with their vancomycin had KIM-1 amounts that were elevated compared to protamine alone as a base comparison (KIM-1 29.0 ng/24 h, 95% CI 5.0–53.0). When comparing each animal group to itself (i.e., change from baseline of day 1), animals that received protamine only did not have a KIM-1 increase over the 4 days (*P* > 0.99), indicating that it was a stable control over the time course of the experiment (Table 2). Animals that received vancomycin alone had elevated KIM-1 on days 2 and 3 (*P* < 0.05) compared to baseline day 1. Animals that received protamine with their vancomycin had KIM-1 elevated compared to baseline day 1 on day 4.

**TABLE 1:** Comparison of KIM-1 across drug treatment groups by day

Drug	Day	Contrast KIM-1 (ng/24 h)	Bonferroni *P* value	(95% confidence interval)
Vancomycin vs protamine alone	1	0.72	>0.99	(−22.3–23.8)
Vancomycin vs protamine alone	2	24.92	0.025	(1.9–48.0)
Vancomycin vs protamine alone	3	20.88	0.106	(−2.2–43.9)
Vancomycin vs protamine alone	4	9.00	>0.99	(−14.0–32.0)
Vancomycin + protamine vs protamine alone	1	0.10	>0.99	(−22.9–23.1)
Vancomycin + protamine vs protamine alone	2	−0.22	>0.99	(−23.3–22.8)
Vancomycin + protamine vs protamine alone	3	13.53	0.868	(−9.5–36.6)
Vancomycin + protamine vs protamine alone	4	29.01	0.008	(5.0–53.0)

**TABLE 2:** Comparison of KIM-1 across days within treatment groups with baseline as a comparator

Drug	Day	Contrast KIM-1 (ng/24 h)	Bonferroni *P* value	(95% confidence interval)
Protamine + vancomycin	2 vs 1	0.29	>0.99	(−19.3–19.8)
Protamine + vancomycin	3 vs 1	13.78	0.457	(−5.8–33.3)
Protamine + vancomycin	4 vs 1	29.19	0.001	(8.5–49.9)
Protamine alone	2 vs 1	0.61	>0.99	(−18.9–20.2)
Protamine alone	3 vs 1	0.35	>0.99	(−19.2–19.9)
Protamine alone	4 vs 1	0.28	>0.99	(−19.3–19.8)
Vancomycin	2 vs 1	24.81	0.004	(5.2–44.4)
Vancomycin	3 vs 1	20.50	0.033	(0.9–40.1)
Vancomycin	4 vs 1	8.55	>0.99	(−11–28.1)

### Plasma iohexol clearance

**Fig 5:**
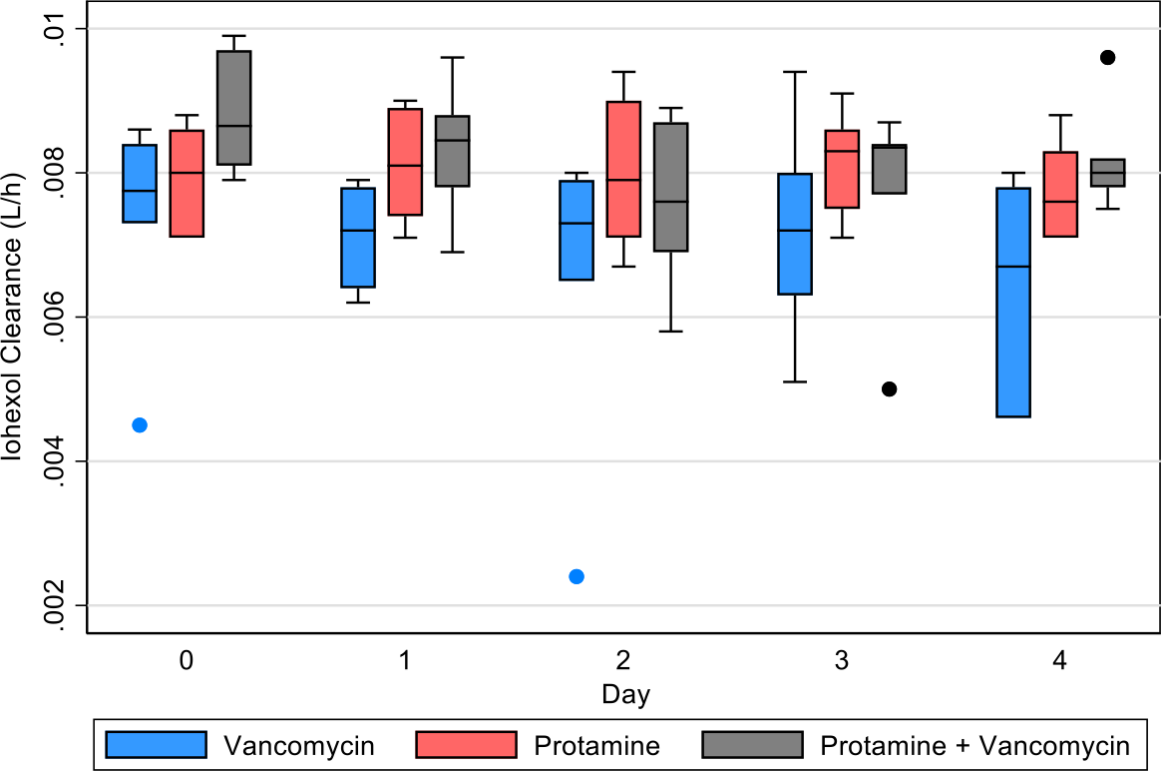
Calculated plasma iohexol clearance for each treatment group that received iohexol.

The omnibus test was only significant for the drug (*P* = 0.047). No statistically observed differences were identified in the Bonferroni corrected results for comparing between drug groups or when comparing clearance change from baseline (Fig. 5, *P* > 0.05).

### Cellular studies

**Fig 6:**
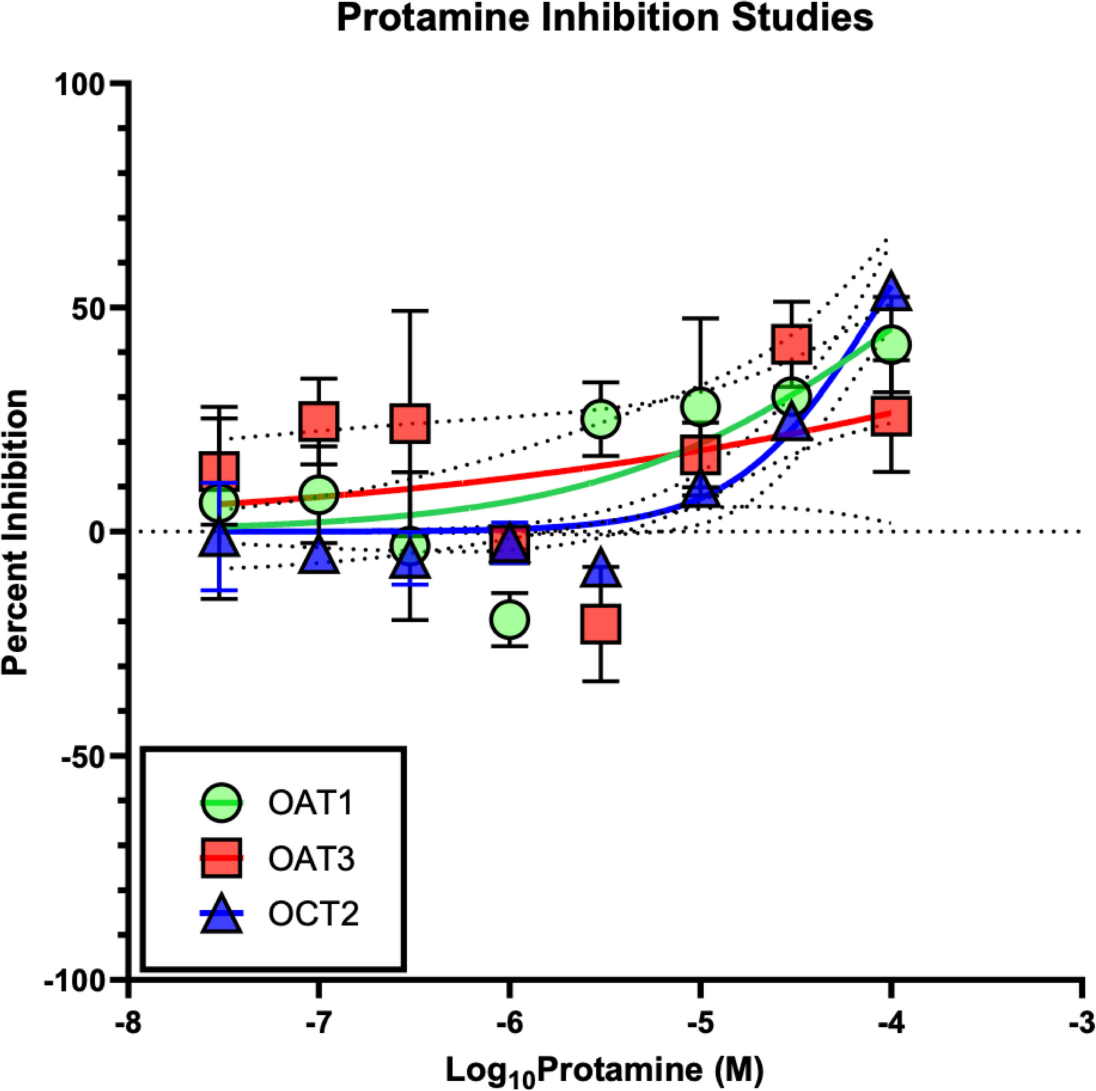
Cellular inhibition studies with protamine, shown in molar (M) concentrations.

Cellular study results are shown in Fig. 6. No substantial inhibition of OAT1, OAT3, or OCT2 was observed. IC_50_ values for protamine were 0.1 mM for OAT1 and OAT3 and 0.043 mM for OCT2. Compared to prototype drugs causing inhibition (i.e., probenecid for OAT1/3 and verapamil for OCT2), IC_50_ values were 62.5 and 58.8 times higher for protamine vs probenecid (i.e., IC_50_s of 0.0016 and 0.0017 mM) and 5.2 times higher for protamine vs verapamil (IC_50_ of 0.0083 mM).

## DISCUSSION

Our results demonstrate that the addition of protamine to vancomycin provides a protective effect against vancomycin-associated nephrotoxicity and that the effect is not likely driven by OAT1, OAT3, or OCT2 inhibition. These results are important as protamine is currently an FDA-approved medication and has the potential to decrease VAN, especially when given immediately prior to short vancomycin courses. These results may warrant a clinical study; however, protamine has a suboptimal safety profile with liabilities of severe hypotension and anaphylactoid-like reactions (40). Additional work is needed to confirm the putative mechanism of activity, i.e., megalin blockade. Drug development for medications with fewer liabilities will be important if the mechanism can be clarified.

In humans, VAN (creatinine defined) (41) occurs between an interquartile range of 6–13 days (42), and creatinine is known to lag behind GFR (43). Serum creatinine is the most commonly utilized biomarker to classify AKI in patients (41, 44–46); however, creatinine is non-sensitive, non-specific, and slowly reactive to kidney function as compared to GFR (47). GFR can drop 50% before changes in SCr are detected (48–50). As such, acute injuries from vancomycin take a median of 6 days to be detectable with creatinine alone (51). Therefore, this study focused on the pre-clinical urinary biomarker, KIM-1, which has been shown as sensitive and specific for vancomycin kidney injury (6, 52). VAN with urinary KIM-1 in the rat model is detectable within a single calendar day (6, 34), and the model links to human outcomes (14). Protamine demonstrates a full day of injury prevention and a delay of 72 h before the mean injury for vancomycin + protamine reaches the vancomycin injury peak seen after 1 day of dosing (Fig. 4). Since ~60% of clinical vancomycin use is ≤2 days (53, 54), delaying toxicity could lower VAN.

Originally derived from salmon fish sperm (salmine), protamine is a highly positively charged protein consisting of 32 amino acids. Currently, it is widely used as an effective agent to neutralize heparin using electrostatic bonds made between the cationic arginine groups of protamine and the anionic heparin in a 1:1 ratio (6). Specifically, protamine shares binding sites with heparin (current target for FDA-approved use) and megalin (30, 31). On this note, a clinical limitation for the use of protamine arises for patients who require heparin infusions, such as post-diabetic foot infection amputation or other surgical interventions. These patients would not be able to use protamine as a VAN protective agent due to the neutralization of heparin. Our preliminary data demonstrate that protamine does not have a significant inhibition on OAT1, OAT3, or OCT2 except at extremely high concentrations (Fig. 6) when compared to known inhibitors such as probenecid and verapamil. Protamine enters kidney epithelial cells and localizes in the cytoplasm via receptor-mediated endocytosis, putatively through megalin, and has demonstrated prevention of gentamicin accumulation (55). Gentamicin is known to share a megalin cell entry mechanism with vancomycin (18, 24, 56). Furthermore, rats and humans share identical megalin extracellular motifs/ligand-binding sites, making the rat ideal for pre-clinical study (57, 58). Our pilot studies with PRO demonstrate a full day of injury prevention and a delay of 72 h before the mean injury for vancomycin + protamine reaches the vancomycin peak seen after 1 day of dosing, though full dose-ranging experiments are required to define the dynamic toxicity threshold. Since ~60% of clinical vancomycin use is ≤2 days (53, 54), delaying toxicity will lower VAN. In humans, VAN (creatinine defined) (41) can occur early after treatment initiation with an interquartile range of 6–13 days (42), and creatinine is known to lag behind GFR (43).

Reabsorption of solutes from glomerular filtrate in the kidneys occurs primarily in the proximal renal tubule (59). Megalin is an endocytic transmembrane protein of the low-density lipoprotein receptor-related protein 2 family (LRP2) (24). It is located in the apical membrane of proximal tubule cells and reabsorbs low molecular weight proteins through endocytosis (24). Beenken et al. (60) used high-resolution cryo-electron microscopy to study LRP2 isolated from mouse kidneys. Endosomes with receptor/ligand complex release ligands intracellularly through lysosome interaction; however, megalin is recycled back to the apical membrane to be used again. They were able to demonstrate that ligand binding and release is pH dependent within extracellular (pH 7.5) and endosomal (pH 5.2) environments. Structurally, at the cell surface, megalin adopts an open conformation exposing the high-affinity ligand binding site at pH 7.5. At endosomal pH 5.2, megalin is transformed into a closed conformation with low-binding affinity, thus releasing the ligand. The endosome with megalin moves back to the apical membrane, where at extracellular pH 7.5, it once again resumes the open, high-affinity conformation to bind ligand. Vancomycin is known to bind to megalin, and its accumulation within proximal tubule cells is nephrotoxic causing oxidative damage and apoptosis (3). Vancomycin, a basic compound, is protonated at urinary pH 7.5 and binds to high-affinity sites on megalin, being released intracellularly in the proximal tubule cell as acidic pH induces the low-affinity conformation.

The exact mechanism of VAN is not entirely known, but current studies hypothesize that damage is directly caused by intracellular accumulation of vancomycin (7). Vancomycin is transited with drug transporters such as OCT-2 located on the basolateral membrane of the proximal renal tubule and inhibits transporters OAT-1 and OAT-3. From the cell studies completed for our research (see Fig. 6), it appears that with protamine, there is little to no inhibition of these transporters. With OAT-1 and OAT-3, inhibition does not reach 50% at 0.1 mM concentrations of protamine. With OCT-2, there does appear to be minor inhibition reaching at least 50% at similar concentrations. This likely means that protamine acts as a weak, potentially non-pharmacologically useful substrate and/or inhibitor at high concentrations.

With the evidence that protamine has little to no interactions with the basolateral membrane transporters OAT-1, OAT-3, and OCT-2, the question still remains of how protamine interacts with vancomycin to delay the onset of AKI when co-administered with vancomycin. It is known from previous studies that vancomycin binds directly to the megalin receptor located within the proximal tubule. Hori et al. (18) performed a study to evaluate the binding of megalin to vancomycin and cisplatin, as well as aminoglycosides and colistin. They utilized quartz crystal microbalance analysis to assess the direct binding of megalin with these drugs and found that megalin is bound specifically by gentamicin, colistin, vancomycin, and cisplatin. Furthermore, they found that the binding of all the drugs listed above is competed by cilastatin.

Despite the promise of protamine as a new treatment approach, this study is subject to several limitations. First, our study was performed in rats. While the rat model has shown strong relevance to the human condition (14) with respect to VAN, clinical trials are necessary to confirm the effect. Second, protamine delayed kidney injury by 1–3 days; however, a multi-pronged approach may be needed to fully suppress injury in vancomycin therapy that exceeds 2 days. Third, as noted above, protamine is not a viable therapeutic for patients who require heparin. Fourth, protamine itself is associated with various toxicities, including cardiopulmonary dysfunction and shock as well as antibody-mediated hypersensitivity reactions (61). Widespread use of protamine may not confer a safer profile to the patient. Despite these liabilities for protamine, the mechanistic approach identified here warrants further study.

### Conclusion

Protamine, when added to vancomycin therapy, delays VAN as defined by urinary KIM-1 in the Sprague-Dawley rat model by 1–3 days. Protamine putatively acts through the blockade of megalin and does not appear to have significant inhibition on OAT1, OAT3, or OCT2. Since protamine is an approved FDA medication, it has clinical potential as a repurposed therapeutic to reduce vancomycin-related kidney injury; however, greater utility may be found by pursuing compounds with fewer adverse event liabilities.

## Data Availability

The data that support the findings of this study are available from the corresponding author upon reasonable request. Data sharing will be subject to standard Data Use Agreements from Midwestern University.
